# An Effective Flux Framework for Linear Irreversible Heat Engines: Case Study of a Thermoelectric Generator

**DOI:** 10.3390/e26030219

**Published:** 2024-02-29

**Authors:** Jasleen Kaur, Ramandeep S. Johal

**Affiliations:** Department of Physical Sciences, Indian Institute of Science Education and Research Mohali, Sector 81 SAS Nagar, Manauli 140306, Punjab, India; ph15054@iisermohali.ac.in

**Keywords:** thermoelectricity, nonequilibrium phenomena, linear irreversible thermodynamics, effective flux

## Abstract

We consider an autonomous heat engine in simultaneous contact with a hot and a cold reservoir and describe it within a linear irreversible framework. In a tight-coupling approximation, the rate of entropy generation is effectively written in terms of a single thermal flux that is a homogeneous function of the hot and cold fluxes. The specific algebraic forms of the effective flux are deduced for scenarios containing internal and external irreversibilities for the typical example of a thermoelectric generator.

## 1. Introduction

In the thermodynamic study of irreversible processes, the rate of entropy generation, $\dot{S}$, is a fundamental quantity. It may be expressed as the sum of the products of flux $J_{\alpha }$ and its associated thermodynamic force $X_{\alpha }$, or $\dot{S}=\sum_{\alpha }J_{\alpha }X_{\alpha }$. For a device that involves energy conversion, such as a heat engine, the description requires at least two flux–force pairs such that

(1) $$\dot{S}=J_{1}X_{1}+J_{2}X_{2}.$$

Here, $J_{2}$ is the spontaneous heat flux ($J_{2}X_{2}>0$), while the velocity flux $J_{1}$ is the driven flux ($J_{1}X_{1}<0$). In order to satisfy the second law, we must have $\dot{S}\geq 0$.

For a generic heat engine in simultaneous contact with two heat reservoirs at temperatures $T_{h}>T_{c}$, let ${\dot{Q}}_{h}$ and ${\dot{Q}}_{c}$ respectively denote the thermal flux entering and exiting the engine. The power output of the device is given by:

(2) $$P={\dot{Q}}_{h}-{\dot{Q}}_{c}=F\dot{x},$$

where *F* is the external load and $\dot{x}$ is the velocity flux. The overall rate of entropy generation is

(3) $$\begin{array}{ccc} \dot{S} & = & \frac{{\dot{Q}}_{c}}{T_{c}}-\frac{{\dot{Q}}_{h}}{T_{h}}. \end{array}$$

In order to express the above in the canonical form, we use (2) to rewrite

(4) $$\dot{S}=\dot{x}\left(-\frac{F}{T_{c}}\right)+{\dot{Q}}_{h}\left(\frac{1}{T_{c}}-\frac{1}{T_{h}}\right),$$

and thus identify the following two flux–force pairs:

(5) $$\begin{array}{cc} J_{1} & =\dot{x},\;X_{1}=-\frac{F}{T_{c}}, \end{array}$$

(6) $$\begin{array}{cc} J_{2} & ={\dot{Q}}_{h},\;X_{2}=\frac{1}{T_{c}}-\frac{1}{T_{h}}, \end{array}$$

The description of irreversible processes is further enriched by assuming small magnitudes for the forces and proposing linear flux–force relations: $J_{\alpha }=\sum_{\beta }L_{\alpha \beta }X_{\beta }$, where $L_{\alpha \beta }$ are the Onsager coefficients, which obey

(7) $$L_{\alpha \alpha }\geq 0,\;L_{\alpha \alpha }L_{\beta \beta }\geq L_{\alpha \beta }^{2},$$

where we assume Onsager reciprocity, $L_{\beta \alpha }=L_{\alpha \beta }$. Now, it is also well known that there is no unique choice of flux–force pairs to express the rate of entropy generation. Thus, for the thermal flux $J_{2}$, one may choose the cold flux ${\dot{Q}}_{c}$. Accordingly, the modified force $X_{1}=F/T_{h}$. Other authors have proposed using a mean thermal flux over the hot and cold fluxes [1,2]. The mean flux also plays a role in the linear irreversible framework for coupled autonomous machines [3]. In these previous approaches, two fluxes in general describe the rate of entropy generation, as in Equation (1). On the other hand, under the so-called strong coupling condition, $L_{\alpha \alpha }L_{\beta \beta }=L_{\alpha \beta }^{2}$, we have the result that $J_{1}$ is directly proportional to $J_{2}$. In this case, only one flux is adequate to describe the rate of entropy generation [4].

However, the above description is often studied within the *local* linear irreversible framework for steady-state heat devices [5]. In this paper, we develop a corresponding approach to the scaled-up *global* framework [6,7,8] for steady-state devices and describe the rate of entropy generation in terms of a single effective flux $\dot{Q}$ and its corresponding generalized force F such that $\dot{S}=\dot{Q}F$. Further, we keep the thermodynamic description within a linear framework, which implies a linear flux–force relation, $\dot{Q}=\lambda F$, where λ is a suitable transport coefficient. So the rate of entropy generation is effectively expressed as:

(8) $$\dot{S}=\frac{{\dot{Q}}^{2}}{\lambda }.$$

Now, the validity of the second law requires that λ>0 [9]. The exact form of $\dot{Q}$ depends on details of the model: in particular, the way irreversibilities are treated within the model. In this paper, we apply our formalism to the Constant Properties Model of a thermoelectric generator (TEG), which provides a paradigmatic model for such a heat engine. In this case, internal and external irreversibilities can be formulated relatively easily [6,10,11,12]. Under various approximations whereby one or the other kind of irreversibility can be neglected, we identify the corresponding effective flux along with the transport coefficient λ for each case.

The paper is organized as follows. In Section 2, we outline the effective flux approach and develop the basic expressions for power output. In Section 3, we describe the so-called Constant Properties Model of a TEG. In Section 4, we determine the effective thermal flux in a TEG with internal irreversibility only, while Section 5 deals exclusively with an external irreversibility. Section 6 is devoted to TEG with both internal and external irreversibilities. Finally, in Section 7, we compare the present framework with the corresponding formalism for discrete heat engines developed by one of the authors [13] and conclude with a discussion of our results.

## 2. The Effective Flux Approach

In the proposed scheme, we assume the validity of Equations (3) and (8). It is convenient to define the ratio $\beta ={\dot{Q}}_{c}/{\dot{Q}}_{h}$. Then, we can express Equation (3) as:

(9) $$\dot{S}=\frac{{\dot{Q}}_{h}}{T_{c}}\left(\beta -\theta \right),$$

where $\theta =T_{c}/T_{h}$. Now, to assign a specific form to the effective flux, we note that ${\dot{Q}}_{c}\leq {\dot{Q}}_{h}$, where the equality implies vanishing power output. The effective flux is bounded by the given hot and cold fluxes: ${\dot{Q}}_{c}\leq \dot{Q}\leq {\dot{Q}}_{h}$. More generally, we demand that $\dot{Q}$ is a homogeneous function of ${\dot{Q}}_{h}$ and ${\dot{Q}}_{c}$ so that we can express $\dot{Q}={\dot{Q}}_{h}q\left(\beta \right)$, where q(β) is to be determined as a function of β.

Thus, Equation (8) can be written as:

(10) $$\dot{S}=\frac{{\dot{Q}}_{h}^{2}q^{2}\left(\beta \right)}{\lambda }.$$

From Equations (9) and (10), we can write:

(11) $${\dot{Q}}_{h}=\frac{\lambda }{T_{c}}\frac{\left(\beta -\theta \right)}{q^{2}\left(\beta \right)}.$$

Finally, power output can be expressed as: $P=\left(1-\beta \right){\dot{Q}}_{h}$. Upon using Equation (11), we obtain

(12) $$P\left(\beta \right)=\frac{\lambda }{T_{c}}\frac{\left(1-\beta )(\beta -\theta \right)}{q^{2}\left(\beta \right)}.$$

Note that apart from q(β), *P* is expressed as a universal expression in terms of β [13,14]. The actual expression for *P* as followed under different models dictates the form taken by the function q(β), which in turn determines the effective flux $\dot{Q}$. We shall illustrate these cases using the TEG model as described in the following.

## 3. TEG Model

**Figure 1:**
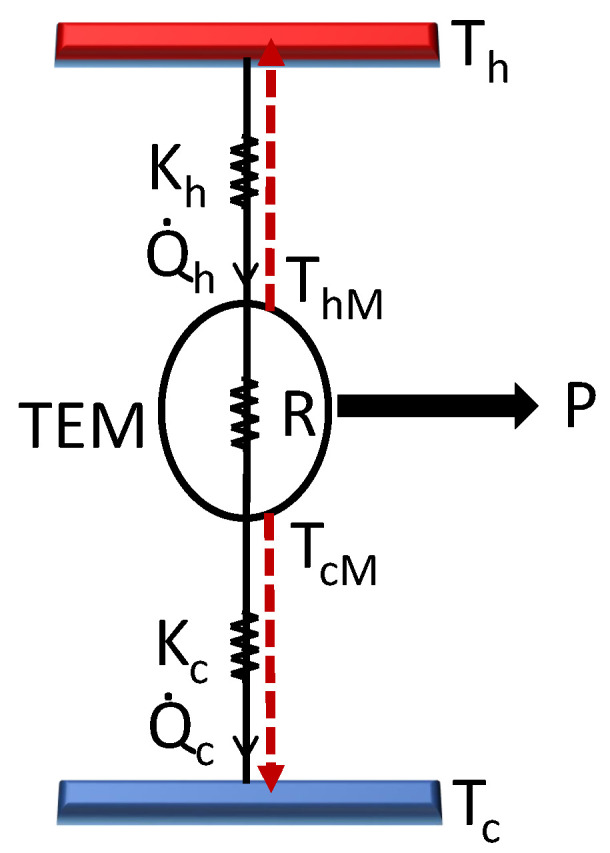
Block diagram of TEG in contact with hot and cold reservoirs via heat exchangers with thermal conductances $K_{h}^{\prime }$ and $K_{c}^{\prime }$. *R* is the internal resistance of TEM with electric current *I* flowing through it. $T_{hM}$ and $T_{cM}$ are the local temperatures of TEM towards the hot and cold side, respectively. Dashed lines indicate flow of Joule heat into each reservoir.

Thermoelectricity is a non-equilibrium phenomenon and can be studied within the framework of Onsager–Callen theory [15,16,17,18]. The coupling between the gradients of temperature and electric potential gives rise to various thermoelectric effects. Consider the thermoelectric material (TEM) to be a one-dimensional substance of length *L* with fixed values of internal resistance *R* and Seebeck coefficient α. Let *I* denote the constant current flowing through the TEM (see Figure 1). Also, we work within the “strong coupling” assumption [19] whereby any heat leakage term between the reservoirs is regarded as negligible compared to the other terms in the heat flux equation. This requires that the thermal conductivity (κ) of the material is very small compared to its electrical conductivity (σ). This is an experimental challenge since the electrical and thermal conductivities ($\kappa_{e}$) of charge carriers are considered proportional to each other, which is encapsulated as the Wiedemann–Franz law. This law has been verified experimentally for a variety of metals and over a wide range of temperatures [20]. Apart from the electronic contribution, there is the lattice part of the thermal conductivity of a material ($\kappa_{l}$) such that $\kappa =\kappa_{e}+\kappa_{l}$. Thus, it is desirable to decrease the latter contribution also in order to diminish the overall thermal conductivity. In recent years, research on topological materials [21,22] has opened up new vistas for material properties that hold promise for the design of thermoelectric materials also. Thus, violations of the Wiedemann–Franz law have been observed in some of these topological materials for which $\kappa_{e}$ can be reduced relative to σ over a certain range of temperatures [23]. Again, the lattice thermal conductivity can be minimized in certain topological insulators [24]. It is with regard to the possibility of using thermoelectric materials with such properties that we formulate and analyze our model.

Then, based on the Onsager formalism and Domenicali’s heat equation [25], thermal fluxes at the end points of TEM may be written as follows [7,8].

(13) $$\begin{array}{ccc} {\dot{Q}}_{h} & = & \alpha T_{hM}I-\frac{1}{2}RI^{2}, \end{array}$$

(14) $$\begin{array}{ccc} {\dot{Q}}_{c} & = & \alpha T_{cM}I+\frac{1}{2}RI^{2}. \end{array}$$

In the above equations, the first term corresponds to convective heat flow, where $T_{hM}$$\left(T_{cM}\right)$ is the local temperature of TEM at the hot (cold) side. The second term is the Joule heat received by each reservoir. Assuming Newtonian heat flow [26,27] between a reservoir and TEM, the hot and cold fluxes are also given by

(15) $$\begin{array}{ccc} {\dot{Q}}_{h} & = & K_{h}^{\prime }\left(T_{h}-T_{hM}\right), \end{array}$$

(16) $$\begin{array}{ccc} {\dot{Q}}_{c} & = & K_{c}^{\prime }\left(T_{cM}-T_{c}\right), \end{array}$$

where $K_{h}^{\prime }$ and $K_{c}^{\prime }$ are the heat transfer coefficients. From the equality of Equations (13) and (15), we get an expression for $T_{hM}$ as:

Substituting this into Equation (15), we can write

(17) $${\dot{Q}}_{h}=K_{h}^{\prime }\frac{2\alpha T_{h}I-RI^{2}}{2(K_{h}^{\prime }+\alpha I)}.$$

Similarly, for the cold flux, we obtain:

(18) $${\dot{Q}}_{c}=K_{c}^{\prime }\frac{2\alpha T_{c}I+RI^{2}}{2(K_{c}^{\prime }-\alpha I)}.$$

Using the definition of β, we can express the electric current as a function of β: I(β). Further, we verify that *I* is a monotonic function of β. Thereby, we express power output in terms of the variable β, as in Equation (12).

## 4. TEG with Internal Irreversibility

In this case, the only source of irreversibility is the internal electrical resistance of the working substance. No dissipation is involved due to thermal contacts with heat reservoirs. More precisely, we consider the limit $K_{h}^{\prime },K_{c}^{\prime }\to \infty$. This leads to the simplification: $T_{hM}=T_{h}$ and $T_{cM}=T_{c}$. Also, we consider bypass or heat leaks to be negligible. This is the so-called exoreversible model [19,28]. So Equations (17) and (18) are simplified to

(19) $$\begin{array}{ccc} {\dot{Q}}_{h} & = & \alpha T_{h}I-\frac{1}{2}RI^{2} \end{array}$$

(20) $$\begin{array}{ccc} \;{\dot{Q}}_{c} & = & \alpha T_{c}I+\frac{1}{2}RI^{2}. \end{array}$$

Now, expressing *I* in terms of β by using Equation (19) and (20), we obtain

(21) $$I=\frac{2\alpha T_{h}}{R}\frac{\left(\beta -\theta \right)}{\left(1+\beta \right)}.$$

So the power output can be expressed in the form

(22) $$P\left(\beta \right)=K_{\mathrm{int}}T_{h}\frac{4(1-\beta )(\beta -\theta )}{{\left(1+\beta \right)}^{2}},$$

where we define the thermal conductance of TEM as [10,11]

(23) $$K_{\mathrm{int}}=\frac{\alpha^{2}\left(T_{h}+T_{c}\right)}{2R}.$$

Comparing the expressions of power in Equations (12) and (22), we identify $\lambda =K_{\mathrm{int}}T_{h}T_{c}$ as the effective thermal conductivity, and q(β)=(1+β)/2. So the effective thermal flux $\dot{Q}={\dot{Q}}_{h}q\left(\beta \right)$ is given by:

(24) $$\dot{Q}=\frac{{\dot{Q}}_{h}+{\dot{Q}}_{c}}{2}.$$

Thus, entropy generation in an exoreversible model may be effectively described by a thermal flux that is the arithmetic mean of the hot and cold fluxes.

## 5. TEG with External Irreversibility

In this case, the sources of irreversibility are heat exchangers having finite thermal conductances, $K_{h}^{\prime }$ and $K_{c}^{\prime }$, on the hot and the cold side, respectively. Also, we assume that TEM has zero internal resistance (R=0). This is the so-called endoreversible approximation [26,27]. Then, the expressions for the heat fluxes (17) and (18) are simplified to

(25) $${\dot{Q}}_{h}=\frac{\alpha K_{h}^{\prime }T_{h}I}{K_{h}^{\prime }+\alpha I},\;{\dot{Q}}_{c}=\frac{\alpha K_{c}^{\prime }T_{c}I}{K_{c}^{\prime }-\alpha I}.$$

Again, from the above expressions, we obtain *I* in terms of the variable β:

(26) $$I=\frac{K_{h}^{\prime }K_{c}^{\prime }}{\alpha }\frac{\left(\beta -\theta \right)}{\left(\theta K_{c}^{\prime }+\beta K_{h}^{\prime }\right)}.$$

Therefore, the output power in terms of β is given by

(27) $$P=K_{\mathrm{ext}}^{\prime }T_{h}\frac{\left(1-\beta )(\beta -\theta \right)}{\beta },$$

where the contact thermal conductance is

(28) $$K_{\mathrm{ext}}^{\prime }=\frac{K_{c}^{\prime }K_{h}^{\prime }}{K_{h}^{\prime }+K_{c}^{\prime }}.$$

Again, a comparison between Equations (12) and (27) suggests that $q\left(\beta \right)=\sqrt{\beta }$, or in other words, the mean thermal flux is the geometric mean:

(29) $$\dot{Q}=\sqrt{{\dot{Q}}_{h}{\dot{Q}}_{c}},$$

with an effective thermal conductivity of

(30) $$\lambda =K_{\mathrm{ext}}^{\prime }T_{h}T_{c}.$$

From Equations (22), (27) and (60), we see that for a thermoelectric generator, the power output can be written in the form of Equation (12), where λ is a constant specific to each irreversibility. More importantly, the effective heat flux has been found to be in the form of either the arithmetic mean or the geometric mean over the hot and cold fluxes. However, so far, we have considered TEG with only one kind of irreversibility. In the following, we find specific configurations with both internal and external irreversibilities and show instances of the effective heat flux in the form of generalized means.

## 6. TEG with Internal and External Irreversibilities

In the Constant Properties Model, Joule heat is distributed equally between hot and cold junctions [8,29]. We first study simpler configurations wherein the external irreversibility is assumed only at one junction while the other junction is perfectly connected with the reservoir. Finally, a configuration with external irreversibility at both junctions is considered. For convenience, we define *v* as the ratio of external to internal thermal conductances:

(31) $$v=\frac{K_{\mathrm{ext}}^{\prime }}{K_{\mathrm{int}}},$$

where $K_{\mathrm{int}}$ and $K_{\mathrm{ext}}^{\prime }$ are defined as in Equations (23) and (28), respectively.

### 6.1. Finite $K_{h}^{\prime }$

Here, the external irreversibility is only at the hot junction due to the finite thermal conductance ($K_{h}^{\prime }$). The cold junction is assumed to be ideal ($K_{c}^{\prime }\to \infty$). Thermal fluxes (17) and (18) are written as

(32) $$\begin{array}{ccc} {\dot{Q}}_{h} & = & K_{h}^{\prime }\frac{2\alpha T_{h}I-RI^{2}}{2(K_{h}^{\prime }+\alpha I)}, \end{array}$$

(33) $$\begin{array}{ccc} {\dot{Q}}_{c} & = & \alpha T_{c}I+\frac{1}{2}RI^{2}. \end{array}$$

The expression of *I* in terms of β is

(34) $$I=\frac{\alpha T_{h}}{4R}\left[-4\theta -v\left(1+\theta \right)\left(1+\beta \right)+\sqrt{{\left\{4\theta +v\left(1+\theta \right)\left(1+\beta \right)\right\}}^{2}+16v\left(1+\theta \right)\left(\beta -\theta \right)}\right],$$

where $v=2RK_{h}^{\prime }/\left[\alpha^{2}T_{h}\left(1+\theta \right)\right]$. Then, the power output can be written as:

(35) $$P\left(\beta \right)=\frac{8\left(1+\theta \right)K_{h}^{\prime }T_{h}\left(1-\beta \right)\left(\beta -\theta \right)}{A\;q^{2}\left(\beta \right)},$$

where $A=4\{2+v\left(1+\theta \right)+\sqrt{4\theta^{2}+v^{2}{\left(1+\theta \right)}^{2}+4v\left(1+\theta \right)}\}$. The effective thermal flux is given by

(36) $$\begin{array}{cc} \dot{Q} & =\frac{1}{A^{1/2}}\left[\left\{v\left(1+\theta \right)-4\theta \right\}{\dot{Q}}_{h}^{2}+v\left(1+\theta \right){\dot{Q}}_{c}^{2}+\left\{4\left(2+\theta \right)+2v\left(1+\theta \right)\right\}{\dot{Q}}_{h}{\dot{Q}}_{c}\right.\\ \; & {\left.\;+2\left({\dot{Q}}_{h}+{\dot{Q}}_{c}\right)\sqrt{4\theta^{2}+v^{2}{\left(1+\theta \right)}^{2}+4v\left(1+\theta \right)}\sqrt{a_{1}{\dot{Q}}_{h}^{2}+a_{2}{\dot{Q}}_{c}^{2}+a_{3}{\dot{Q}}_{c}{\dot{Q}}_{h}}\right]}^{1/2}, \end{array}$$

where

(37) $$\begin{array}{ccc} a_{1} & = & \frac{v^{2}{\left(1+\theta \right)}^{2}+16\theta^{2}-8v\theta \left(1+\theta \right)}{4[4\theta^{2}+v^{2}{\left(1+\theta \right)}^{2}+4v\left(1+\theta \right)]}, \end{array}$$

(38) $$\begin{array}{ccc} a_{2} & = & \frac{v^{2}{\left(1+\theta \right)}^{2}}{4[4\theta^{2}+v^{2}{\left(1+\theta \right)}^{2}+4v\left(1+\theta \right)]}, \end{array}$$

and $a_{3}=1-a_{1}-a_{2}$. Note that the coefficients have been defined so that $a_{1}+a_{2}+a_{3}=1$. The effective flux has been written in the particular form above to make it clear that $\dot{Q}$ lies between ${\dot{Q}}_{h}$ and ${\dot{Q}}_{c}$. For the limiting cases, when v→0 (physically, R→0), $\dot{Q}\to \sqrt{{\dot{Q}}_{h}{\dot{Q}}_{c}}$, yielding the effective flux corresponding to the endoreversible approximation. On the other hand, when v→∞, implying $K_{h}^{\prime }\to \infty$ at finite *R*, $\dot{Q}\to \left({\dot{Q}}_{h}+{\dot{Q}}_{c}\right)/2$, corresponding to the exoreversible approximation. The effective thermal conductance λ for this case is given by

(39) $$\lambda =\frac{2K_{h}^{\prime }T_{h}T_{c}\left(1+\theta \right)}{2+v\left(1+\theta \right)+\sqrt{4\theta^{2}+v^{2}{\left(1+\theta \right)}^{2}+4v\left(1+\theta \right)}}.$$

For the limiting cases, λ reduces to $K_{h}^{\prime }T_{h}T_{c}$ as v→0 and to $\alpha^{2}\left(T_{h}+T_{c}\right)T_{h}T_{c}/2R$ as v→∞, respectively.

### 6.2. Finite $K_{c}^{\prime }$

In this case, thermal contact at the hot junction is perfect ($K_{h}^{\prime }\to \infty$), and the cold junction has finite thermal conductance $K_{c}^{\prime }$. Then, the thermal fluxes (17) and (18) are given as

(40) $$\begin{array}{ccc} {\dot{Q}}_{h} & = & \alpha T_{h}I-\frac{1}{2}RI^{2}, \end{array}$$

(41) $$\begin{array}{ccc} {\dot{Q}}_{c} & = & K_{c}^{\prime }\frac{2\alpha T_{c}I+RI^{2}}{2(K_{c}^{\prime }-\alpha I)}. \end{array}$$

The expression for *I* in terms of β is

(42) $$I=\frac{\alpha T_{h}}{4R\beta }\left[4\beta +v\left(1+\beta \right)\left(1+\theta \right)-\sqrt{{\left\{4\beta +v\left(1+\beta \right)\left(1+\theta \right)\right\}}^{2}-16v\beta \left(1+\theta \right)\left(\beta -\theta \right)}\right],$$

where $v=2RK_{c}^{\prime }/\left\{\alpha^{2}T_{h}\left(1+\theta \right)\right\}$. From the expression of the power output, the normalized effective thermal flux is found to be

(43) $$\begin{array}{cc} \dot{Q} & =\frac{1}{B^{1/2}}\left[v\left(1+\theta \right){\dot{Q}}_{h}^{2}+\left\{v\left(1+\theta \right)-4\right\}{\dot{Q}}_{c}^{2}+\left\{4\left(1+2\theta \right)+2v\left(1+\theta \right)\right\}{\dot{Q}}_{h}{\dot{Q}}_{c}\right.\\ \; & {\left.\;+2\left({\dot{Q}}_{h}+{\dot{Q}}_{c}\right)\sqrt{4+v^{2}{\left(1+\theta \right)}^{2}+4v\theta \left(1+\theta \right)}\sqrt{b_{1}{\dot{Q}}_{h}^{2}+b_{2}{\dot{Q}}_{c}^{2}+b_{3}{\dot{Q}}_{h}{\dot{Q}}_{c}}\right]}^{1/2}, \end{array}$$

where $B=4\{v\left(1+\theta \right)+2\theta +\sqrt{4+v^{2}{\left(1+\theta \right)}^{2}+4v\theta \left(1+\theta \right)}\}$ and

(44) $$\begin{array}{ccc} b_{1} & = & \frac{v^{2}{\left(1+\theta \right)}^{2}}{4[4+v^{2}{\left(1+\theta \right)}^{2}+4v\theta \left(1+\theta \right)]}, \end{array}$$

(45) $$\begin{array}{ccc} b_{2} & = & \frac{v^{2}{\left(1+\theta \right)}^{2}+16-8v\left(1+\theta \right)}{4[4+v^{2}{\left(1+\theta \right)}^{2}+4v\theta \left(1+\theta \right)]}, \end{array}$$

where $b_{3}=1-b_{1}-b_{2}$. For the limiting cases, $\dot{Q}$ reduces to the effective thermal flux in the case of endoreversible (v→0) and exoreversible (v→∞) models. The effective thermal conductance equals

(46) $$\lambda =\frac{2K_{c}^{\prime }T_{h}T_{c}\left(1+\theta \right)}{v\left(1+\theta \right)+2\theta +\sqrt{4+v^{2}{\left(1+\theta \right)}^{2}+4v\theta \left(1+\theta \right)}},$$

which goes to $K_{c}^{\prime }T_{h}T_{c}$ and $\alpha^{2}\left(T_{h}+T_{c}\right)T_{h}T_{c}/2R$, respectively, in the above limits.

### 6.3. Finite $K_{c}^{\prime }$ and $K_{h}^{\prime }$

In this case, both external irreversibilities are present simultaneously with internal irreversibility. For simplicity, we set $K_{h}^{\prime }=K_{c}^{\prime }=K^{\prime }/2$. Thermal fluxes on either side are given as

(47) $${\dot{Q}}_{h}=K^{\prime }\frac{2\alpha T_{h}I-RI^{2}}{2(K^{\prime }+2\alpha I)},\;{\dot{Q}}_{c}=K^{\prime }\frac{2\alpha T_{c}I+RI^{2}}{2(K^{\prime }-2\alpha I)}.$$

Now expressing *I* in terms of β, we obtain:

(48) $$\begin{array}{cc} I & =\frac{\alpha T_{h}}{2R}[\frac{\sqrt{8v\left(1+\theta \right)\left(1-\beta \right)\left(\beta -\theta \right)+{\left\{v\left(1+\theta \right)\left(1+\beta \right)+2\left(\beta +\theta \right)\right\}}^{2}}}{\left(1-\beta \right)}\\ \; & \;-\frac{v(1+\theta )(1+\beta )+2(\beta +\theta )}{\left(1-\beta \right)}], \end{array}$$

where $v=RK^{\prime }/\left\{2\alpha^{2}T_{h}\left(1+\theta \right)\right\}$. From the expression for power in terms of β, we can identify the effective mean as:

(49) $$\begin{array}{cc} \dot{Q} & =\frac{1}{C^{1/2}}\left[\left(v+v\theta -2\theta \right){\dot{Q}}_{h}^{2}+\left(v+v\theta -2\right){\dot{Q}}_{c}^{2}+2\left(1+\theta \right)\left(v+3\right){\dot{Q}}_{h}{\dot{Q}}_{c}\right.\\ \; & {\left.\;+2\left({\dot{Q}}_{c}+{\dot{Q}}_{h}\right)\left(v+1\right)\left(1+\theta \right)\sqrt{c_{1}{\dot{Q}}_{h}^{2}+c_{2}{\dot{Q}}_{c}^{2}+c_{3}{\dot{Q}}_{h}{\dot{Q}}_{c}}\right]}^{1/2}, \end{array}$$

where C=8(v+1)(1+θ), and

(50) $$\begin{array}{ccc} c_{1} & = & \frac{v(1+\theta )-2\theta }{4{\left(v+1\right)}^{2}{\left(1+\theta \right)}^{2}}, \end{array}$$

(51) $$\begin{array}{ccc} c_{2} & = & \frac{v(1+\theta )-2}{4{\left(v+1\right)}^{2}{\left(1+\theta \right)}^{2}}, \end{array}$$

where $c_{3}=1-c_{1}-c_{2}$. As expected, for v→0 (R→ 0), $\dot{Q}$ reduces to $\sqrt{{\dot{Q}}_{h}{\dot{Q}}_{c}}$, and for v→∞ ($K^{\prime }\to \infty$), $\dot{Q}\to \left({\dot{Q}}_{h}+{\dot{Q}}_{c}\right)/2$ corresponding to the exoreversible model. We can identify λ in this case as

(52) $$\lambda =\frac{K^{\prime }T_{h}T_{c}}{4(v+1)}.$$

**Figure 2:**
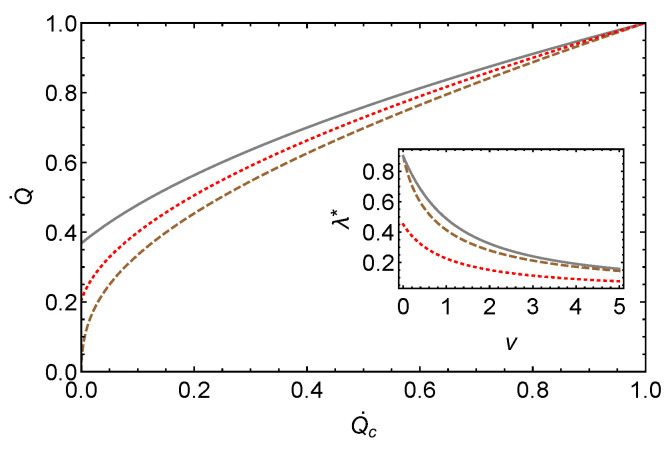
Variation of the effective thermal flux, with cold flux (measured in watts) as in Equation (36) shown by dashed line, as in Equation (43) shown by solid line, and as in Equation (49) by a dotted line, where the parameters are set to v=1, θ=0.5, ${\dot{Q}}_{h}=1\;\mathrm{Watt}$. The insert depicts the effective thermal conductance versus *v* in the corresponding cases $T_{h}=300\;K,T_{c}=150\;K$ and $K_{h}^{\prime }=K_{c}^{\prime }=K^{\prime }=6\times 10^{-3}\;\mathrm{Watt}/K$.

The three effective fluxes derived in this section are depicted in Figure 2, which shows the cold flux for a given hot flux.

## 7. Discussion

The main motivation for this work was to express the rate of entropy generation for a steady-state heat engine under the tight-coupling assumption in terms of a single effective flux. In the standard description of linear irreversible engines within the Onsager framework, the tight-coupling assumption yields a single flux to describe the rate of entropy generation. This applies to steady-state engines at the *local* level. However, a similar analysis has not been carried out when we scale up this description to the *global* level, such as for thermoelectric generators for which the length of the thermoelectric material is finite [6,7,8,28]. It has been observed earlier that thermodynamic forces in the global picture are discrete versions of the local forces. Secondly, the thermal fluxes involve quadratic terms that express Joule heating. This leads to nonlinear flux–force relations. Analogous to the strong-coupling framework at the local level, we formulated entropy generation in terms of the effective thermal flux. We observed explicit forms of this flux in TEG, which is a homogeneous function of the hot and cold fluxes. In the simple case of only external (internal) irreversibility, we obtain the effective flux in the form of the arithmetic (geometric) mean of hot and cold fluxes.

For the endoreversible approximation, we chose Newton’s law, which yields the geometric mean flux. We note that the form of the mean depends on the specific heat transfer law. If instead of Newton’s law, we choose the inverse temperature law of linear irreversible thermodynamics, then we have

(53) $$\begin{array}{ccc} {\dot{Q}}_{h} & = & K_{h}\left(T_{hM}^{-1}-T_{h}^{-1}\right), \end{array}$$

(54) $$\begin{array}{ccc} {\dot{Q}}_{c} & = & K_{c}\left(T_{c}^{-1}-T_{cM}^{-1}\right), \end{array}$$

where $K_{h},K_{c}$ are the heat transfer coefficients. The above fluxes are to be respectively matched with ${\dot{Q}}_{h}=\alpha T_{hM}I$ and ${\dot{Q}}_{c}=\alpha T_{cM}I$, from Equations (13) and (14). Thus, we obtain

(55) $$\begin{array}{ccc} T_{hM} & = & \frac{-K_{h}+\sqrt{K_{h}^{2}+4\alpha K_{h}T_{h}^{2}I}}{2\alpha T_{h}I}, \end{array}$$

(56) $$\begin{array}{ccc} T_{cM} & = & \frac{K_{c}-\sqrt{K_{c}^{2}-4\alpha K_{c}T_{c}^{2}I}}{2\alpha T_{c}I}. \end{array}$$

Therefore, the thermal fluxes entering and exiting the thermoelectric material are as follows [30,31].

(57) $$\begin{array}{ccc} {\dot{Q}}_{h} & = & \frac{\sqrt{K_{h}^{2}+4\alpha K_{h}T_{h}^{2}I}-K_{h}}{2T_{h}}, \end{array}$$

(58) $$\begin{array}{ccc} {\dot{Q}}_{c} & = & \frac{K_{c}-\sqrt{K_{c}^{2}-4\alpha K_{c}T_{c}^{2}I}}{2T_{c}}. \end{array}$$

Now, by using Equations (57) and (58), we express *I* in terms of β as

(59) $$I=\frac{\beta K_{c}K_{h}\left(\beta -\theta \right)\left(\beta K_{h}\theta +K_{c}\right)}{\alpha T_{c}^{2}{\left(K_{c}+\beta^{2}K_{h}\right)}^{2}}.$$

The output power in terms of β is as follows.

(60) $$P\left(\beta \right)=\frac{K_{\mathrm{ext}}}{T_{c}}\frac{\left(1-\beta )(\beta -\theta \right)}{\left(\delta +\left(1-\delta \right)\beta^{2}\right)}$$

where $K_{\mathrm{ext}}=K_{c}K_{h}/\left(K_{h}+K_{c}\right)$ and $\delta =K_{c}/\left(K_{h}+K_{c}\right)$. From Equations (12) and (60), we have $q\left(\beta \right)={\left(\delta +\left(1-\delta \right)\beta^{2}\right)}^{1/2}$, which defines the so-called weighted quadratic mean:

(61) $$\dot{Q}={\left(\delta {\dot{Q}}_{h}^{2}+\left(1-\delta \right){\dot{Q}}_{c}^{2}\right)}^{1/2},$$

with an effective thermal conductance of $\lambda =K_{\mathrm{ext}}$. We also note that as the cold (hot) contact approaches the reversible limit, implying $K_{c}\to \infty \left(K_{h}\to \infty \right)$ or δ→1(δ→0), the effective heat flux goes to the limit ${\dot{Q}}_{h}\left({\dot{Q}}_{c}\right)$.

An analogous effective framework was proposed for discrete heat engines running in a finite cycle period τ [13]. We briefly summarize the important features of this framework. Let the total work extracted be given as $W=Q_{h}-Q_{c}$, and the total entropy produced is $\Delta S=Q_{c}/T_{c}-Q_{h}/T_{h}$. The average power is defined as P=W/τ, and the average rate of entropy production is ΔS/τ. Again, we work in the strong-coupling regime. Instead of effective flux, we postulate effective heat flowing through the system $\bar{Q}$ in time τ. This leads to defining an average effective flux as $\bar{Q}/\tau$. Now, the total rate of entropy production is considered as a quadratic function of the effective flux.

(62) $$\tau =\frac{{\bar{Q}}^{2}}{\lambda \Delta_{\mathrm{tot}}S}$$

In the case of discrete heat devices, the effective heat $\bar{Q}$ may be taken in the form of a homogeneous function of $Q_{h}$ and $Q_{c}$. In contrast, for the case of autonomous devices, we note that it is the effective *flux* that is given in terms of the hot and cold fluxes exchanged with the reservoirs. Despite this difference, the form of the objective function, for instance, the power output, is given by a similar expression in the formulation for discrete engines [13] and the present framework. Thus, for the discrete models, we have

(63) $$P\left(\nu \right)=\frac{\lambda }{T_{c}}\frac{\left(1-\nu )(\nu -\theta \right)}{q^{2}\left(\nu \right)}$$

where $\nu =Q_{c}/Q_{h}$, and so the efficiency η=1−ν. Further, the effective heat is given by $\bar{Q}=Q_{h}q\left(\nu \right)$. The formulation proposed in this paper is applicable to scaled-up steady-state engines. Using the model example of TEG, we are able to analyze different types of irreversibilities that yield specific forms of the effective fluxes. This leads to a simplified description of these models for engines under tight-coupling conditions. A similar analysis may be carried out for a thermoelectric cooler or pump.

## Data Availability

No new data were created or analyzed in this study. Data sharing is not applicable to this article.

## Abbreviations

The following abbreviations are used in this manuscript:

TEG	Thermoelectric Generator
TEM	Thermoelectric Material
